# Quantifying song behavior in a free‐living, light‐weight, mobile bird using accelerometers

**DOI:** 10.1002/ece3.8446

**Published:** 2022-01-23

**Authors:** Elena Eisenring, Marcel Eens, Jean‐Nicolas Pradervand, Alain Jacot, Jan Baert, Eddy Ulenaers, Michiel Lathouwers, Ruben Evens

**Affiliations:** ^1^ Department of Biology Behavioural Ecology and Ecophysiology Group University of Antwerp Wilrijk Belgium; ^2^ Swiss Ornithological Institute Field Station Valais Sion Switzerland; ^3^ Terrestrial Ecology Unit Department of Biology Ghent University Ghent Belgium; ^4^ Agentschap Natuur en Bos Regio Noord‐Limburg Brussels Belgium; ^5^ Research Group: Zoology, Biodiversity and Toxicology Centre for Environmental Sciences Hasselt University Diepenbeek Belgium; ^6^ Department of Geography Institute of Life, Earth and Environment (ILEE) University of Namur Namur Belgium; ^7^ Max Planck Institute for Ornithology Seewiesen Germany

**Keywords:** audio recordings, behavior classification, bioacoustics, biologging, birdsong, *Caprimulgus europaeus*, European nightjar, telemetry, vocalizations

## Abstract

To acquire a fundamental understanding of animal communication, continuous observations in a natural setting and at an individual level are required. Whereas the use of animal‐borne acoustic recorders in vocal studies remains challenging, light‐weight accelerometers can potentially register individuals’ vocal output when this coincides with body vibrations. We collected one‐dimensional accelerometer data using light‐weight tags on a free‐living, crepuscular bird species, the European Nightjar (*Caprimulgus europaeus*). We developed a classification model to identify four behaviors (rest, sing, fly, and leap) from accelerometer data and, for the purpose of this study, validated the classification of song behavior. Male nightjars produce a distinctive “churring” song while they rest on a stationary song post. We expected churring to be associated with body vibrations (i.e., medium‐amplitude body acceleration), which we assumed would be easy to distinguish from resting (i.e., low‐amplitude body acceleration). We validated the classification of song behavior using simultaneous GPS tracking data (i.e., information on individuals’ movement and proximity to audio recorders) and vocal recordings from stationary audio recorders at known song posts of one tracked individual. Song activity was detected by the classification model with an accuracy of 92%. Beyond a threshold of 20 m from the audio recorders, only 8% of the classified song bouts were recorded. The duration of the detected song activity (i.e., acceleration data) was highly correlated with the duration of the simultaneously recorded song bouts (correlation coefficient = 0.87, *N* = 10, *S* = 21.7, *p *= .001). We show that accelerometer‐based identification of vocalizations could serve as a promising tool to study communication in free‐living, small‐sized birds and demonstrate possible limitations of audio recorders to investigate individual‐based variation in song behavior.

## Graphical abstract

We used light‐weight accelerometers to register the vocal output of European Nightjars (*Caprimulgus europaeus*) via body vibrations. We developed a classification model to identify four behaviors (rest, sing, fly, and eat) from accelerometer data and used audio recordings to validate the classification of song behavior. We show that accelerometer‐based identification of vocalizations could serve as a promising tool to study communication in free‐living, small‐sized birds and demonstrate possible limitations of audio recorders to investigate individual‐based variation in song behavior.

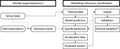

## 1 INTRODUCTION

Animal‐borne tags for behavioral studies are opening a wide range of possibilities to unravel previously undiscovered aspects of animal life (Brown et al., 2013; Johnson et al., 2009; Kays et al., 2015; Nuijten et al., 2020). During the last two decades, a wide range of sensors have been deployed to directly record animals’ position (GPS, passive and active transponder tags), body movements (accelerometers), and internal state (e.g., heart rate and body temperature sensors), as well as the physical environment (e.g., temperature loggers, light sensors, and pressure sensors). These tools enable the automatic collection of individual‐based data on the behavior of free‐living animals over extended periods (Greif & Yovel, 2019; Hughey et al., 2018).

Animal‐borne devices have been widely applied to study many aspects of animal behavior (Kays et al., 2015), but have only rarely been used to study vocalization and song behavior in free‐roaming animals (Greif & Yovel, 2019). It is, however, essential to acquire observations at the individual level in order to gain fundamental insights in animal communication (Gill et al., 2016). Collecting individuals’ vocal activity is challenging when using stationary or handheld bioacoustic recorders, which are mainly suitable to monitor site‐specific vocal activity that may integrate multiple individuals. On the other hand, animal‐borne acoustic recorders are energy consuming and produce large volumes of data, requiring a high storing capacity (Brown et al., 2013; Gill et al., 2016; Greif & Yovel, 2019; Hughey et al., 2018; Korpela et al., 2020). The size, weight, and limited recording duration (~24 h, Couchoux et al., 2015; Cvikel, Levin, et al., 2015) form important restrictions to their use beyond laboratory settings. Several studies have tried to overcome these challenges by directly transmitting the recorded data via low‐power frequency modulation (Anisimov et al., 2014; Gill et al., 2015; Maat et al., 2014) or Bluetooth low energy (Magno et al., 2020) to a receiver; a technique called microphone telemetry. These recent improvements have resulted in light, miniature microphones with extended recording possibilities (up to 10 days or longer) which can be deployed on animals as small as zebra finches (*Taeniopygia guttata*) (Gill et al., 2015, 2016; Magno et al., 2020). However, the restricted transmission distance (a few tens of meters; Gill et al., 2016) still limits the applicability of these systems to study free‐roaming animals.

Less than a decade ago, Goldbogen et al. (2014) and Anisimov et al. (2014) described that animal‐borne accelerometers can register body vibrations that reflect the vocal output of baleen whales (Mysticeti) and zebra finches, respectively. The lower costs, energy consumption, and required storing capacity of accelerometers (Brown et al., 2013; Hughey et al., 2018; Korpela et al., 2020) result in lower weight and smaller‐sized devices with extended logging durations (several days or weeks; Brown et al., 2013). Nonetheless, further investigation of the use of accelerometers in the study of vocal communication has received limited attention (Naito et al., 2010; Oestreich et al., 2020; Saddler et al., 2017; Stimpert et al., 2020; Wijers et al., 2020) and has—to the best of our knowledge—only been applied in two species of bustards which perform booming calls, associated with excessive head movements (little bustards *Tetrax*, Gudka et al., 2019; African houbara bustards *Chlamydotis undulata*, Alonso et al., 2021). The limited adoption of accelerometers in vocal studies is possibly due to the difficulty of assigning accelerometer data to different behaviors in free‐roaming animals (Alonso et al., 2021; Brown et al., 2013; Nathan et al., 2012; Shamoun‐Baranes et al., 2012). Observing the behavior of captive conspecifics for the validation of accelerometer data can circumvent the necessity to make field observations of free‐living individuals, yet may fail to reliably distinguish between different behaviors in free‐roaming populations (Pagano et al., 2017). A particularly challenging species’ group for vocal studies consists of highly mobile, medium‐ and small‐sized birds which often have complex vocalizations. Smaller‐sized birds are only able to carry relatively small devices (Fiore et al., 2017; Magno et al., 2020; Vandenabeele et al., 2012), and the validation of accelerometer‐derived behaviors is often complicated by poor accessibility/detectability of individuals in the wild.

**FIGURE 1:**
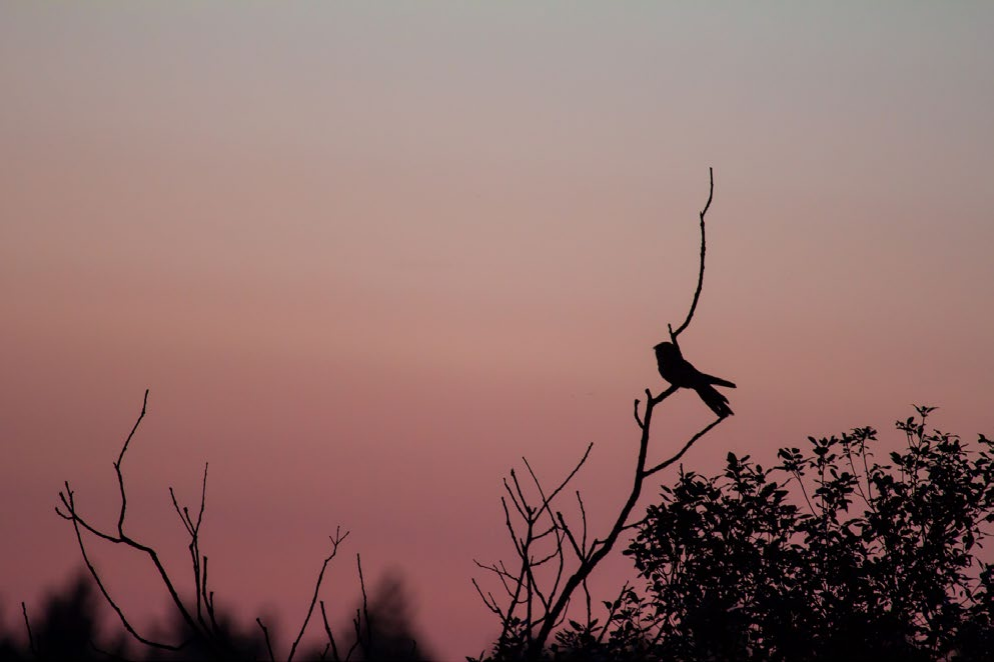
The European Nightjar (*Caprimulgus europaeus*) is a crepuscular insectivore that performs a simple “churring” song, comprising extended repetitive trills from widely distributed stationary song posts

We investigated the usefulness of accelerometers to record the song activity of European Nightjars (*Caprimulgus europaeus*, hereafter nightjar; Figure 1). Nightjars are light‐weight (~70 g; Schlegel, 1967) crepuscular insectivores, which mainly communicate using in‐flight wing‐clapping, “dweep” calls and simple “churring” song. The “churring” song is less complex than many other bird songs and comprises extended repetitive trills, from widely distributed stationary song posts (Rebbeck et al., 2001; Zwart et al., 2014) situated up to several kilometers from the territory center (Evens, Beenaerts, Ulenaers, et al., 2018). Assuming the “churring” song would produce detectable body vibrations, we deployed a custom combination of an accelerometer and a GPS logger on male nightjars and simultaneously made audio recordings at the song posts of tagged males. We first trained a hidden Markov model (HMM) to classify four main behaviors (flying, resting, leaping, and singing) based on one‐dimensional (*Z*‐axis) accelerometer data. We then validated this classification using recorded song behavior from one individual for one night at two different song posts. Based on this exercise, we highlight the potential of accelerometer‐based identification of song activity of individual nightjars as a promising model to study communication in free‐living, small‐sized birds and demonstrate possible limitations of audio recorders in capturing individual variation in song behavior.

## 2 MATERIAL AND METHODS

### 2.1 General field practices

During the 2019 breeding season, we collected acceleration data in various sites in Belgium and Switzerland. All sites were well known from previous studies (Evens, Beenaerts, Neyens, et al., 2018; Evens et al., 2020). Behavioral classification using accelerometer data was also based on the data collected in these two countries. When focusing on the validation of song activity from accelerometer data, we only used data collected in two Belgian sites (Klein Schietveld [N: 51.35, E: 4.49] and Kalmthoutse Heide [N: 51.39, E: 4.43]). Here we also made automated audio recordings at known song posts of tagged individuals using a maximum of 35 SongMeters (SongMeter™ SM2+; Wildlife Acoustics Inc.). The SongMeters were programmed to record environmental sounds at the same time schedule as the accelerometers (for detailed schedules, see further) and recordings (time recorded in GMT) were saved to a 64GB SD card.

We captured nightjars using ultrafine mist nets (Ecotone, 12 × 3 m) and tape lures within presumed territories (Evens et al., 2017) and fitted a custom combination of an accelerometer (0.9 g; SOI‐GDL3), a radio tag (0.4 g; Biotrack Ltd.), and a GPS logger (1.8 g; Pathtrack Ltd.) to the tail of males using a simple drop‐off mechanism (Evens, Beenaerts, Ulenaers, et al., 2018). Tags weighed approximately 4.8 ± 0.3% ([4.4%–5.4%]; total tag weight = 3.1 g) of the mean weight of tagged birds (66 ± 4.7 g, [57–70.9 g]; Appendix S1). We programmed the accelerometers to start measuring one‐dimensional acceleration (*g*, *Z*‐axis, time recorded in GMT) continuously at 25 Hz; from before sunset (9 PM) until after sunrise (6 AM). This allowed the logging of individuals’ activity for a maximum of 48 h. To obtain a value for dynamic acceleration (acceleration resulting from movement), we smoothed the acceleration data using a running mean (2‐s interval) and subtracted the smoothed data from the unsmoothed data to remove the static acceleration (i.e., acceleration resulting from the tag's angle with respect to the Earth's gravity; Nathan et al., 2012). We programmed GPS loggers to fix positions at 3‐min intervals during the same period of the night. For the purpose of this study, we deployed eight loggers and recovered seven loggers in Kalmthoutse Heide and Klein Schietveld. All the recovered loggers dropped from the birds after approximately 6 days (7 ± 3 days, [3–10 days], *n* = 7). From visual inspection of GPS tracks and individuals that were recaptured later in the season, we could not observe abnormal behavior or apparent negative effects from carrying the custom tracking devices.

### 2.2 Behavior classification

**FIGURE 2:**
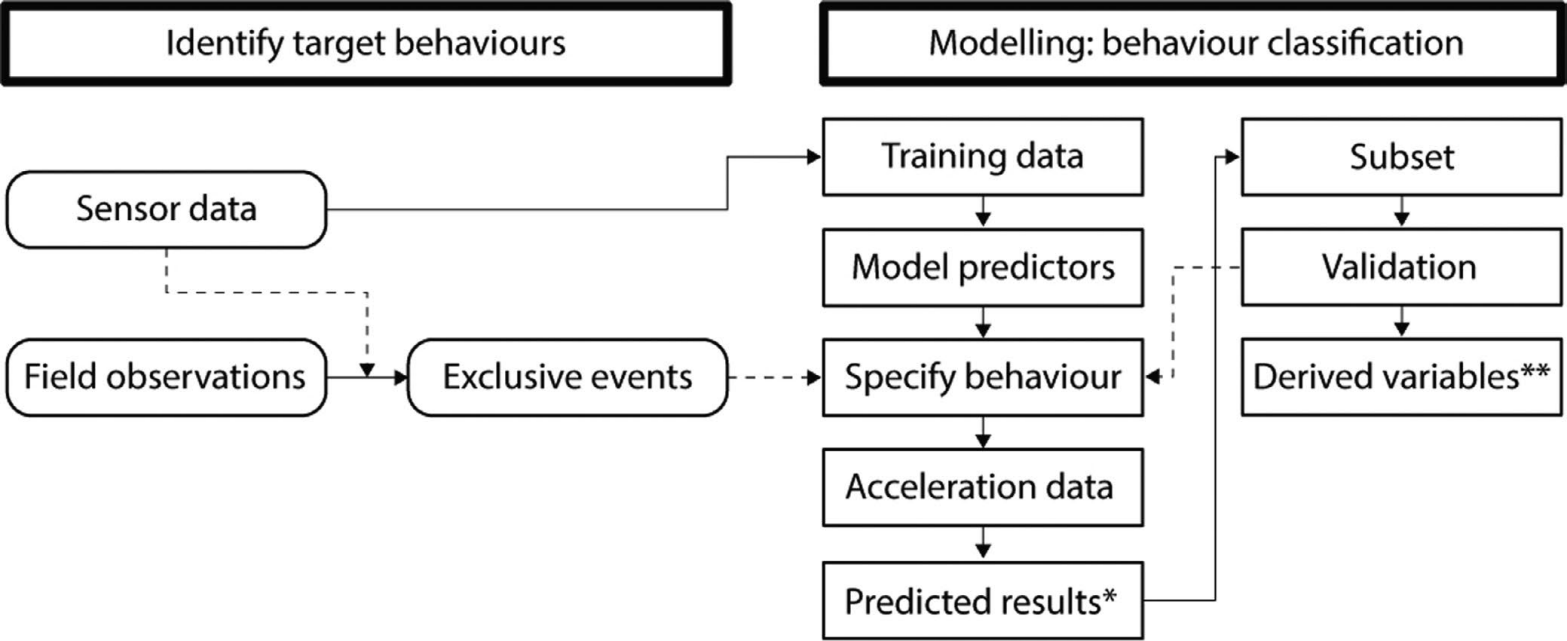
A schematic of the methodological workflow followed in our study to classify behavior from one‐dimensional accelerometer data. The workflow contains two main categories: Identify target behaviors and Modeling. Ovals represent steps involved in data management and rectangles represent steps involved in building of the classification model. Solid arrows present the workflow to move from various data sources to processed data, training the classification model, and finally the application of the classification model to all accelerometer data and the extraction of variables for analyses. Dashed arrows present (i) steps wherein specific information was inserted into the workflow or (ii) feedback loops where a certain part of the workflow is repeated in response to progressive insights. *, Classification of behavior. **, derived variables used as input for generalized linear mixed models

We followed a two‐step approach to classify four behaviors from one‐dimensional accelerometer data (Figure 2). First, we designed an ethogram in order to describe four main behaviors nightjars perform at night: rest, sing, fly, and leap (Table 1). We processed GPS‐based movement data and collected field observations (audio recordings and thermal videos (Videos 2 and 3) of each behavior. Second, we applied unsupervised machine learning to classify accelerometer data and verified the classification with field observations to establish that each of the four behaviors was associated with a distinctive accelerometer signal. For the purpose of this study, we focus on the validation of song activity.

**TABLE 1:** Ethogram of target behaviors

Behavior	Locomotion	Description	GPS observation	Verification
Rest	No	Standing or sitting	Clustered, daytime	Visual observations
Sing	No	Singing	Clustered, breeding habitat	Song recordings
Fly	Yes	Flying	Scattered observations	Inbound commuting flights (Video 2)
Leap	Yes	Chasing prey	Clustered, foraging habitat	Thermal videos (Video 3)

Note

Exclusive events of the four behaviors were identified from GPS observations, validated using various types of field observations, and linked with accelerometer measurements. GPS observation: type of GPS observation used for the identification of behavior. Verification: information/method used to validate the GPS observations.

#### 2.2.1 Identification of target behavior

**FIGURE 3:**
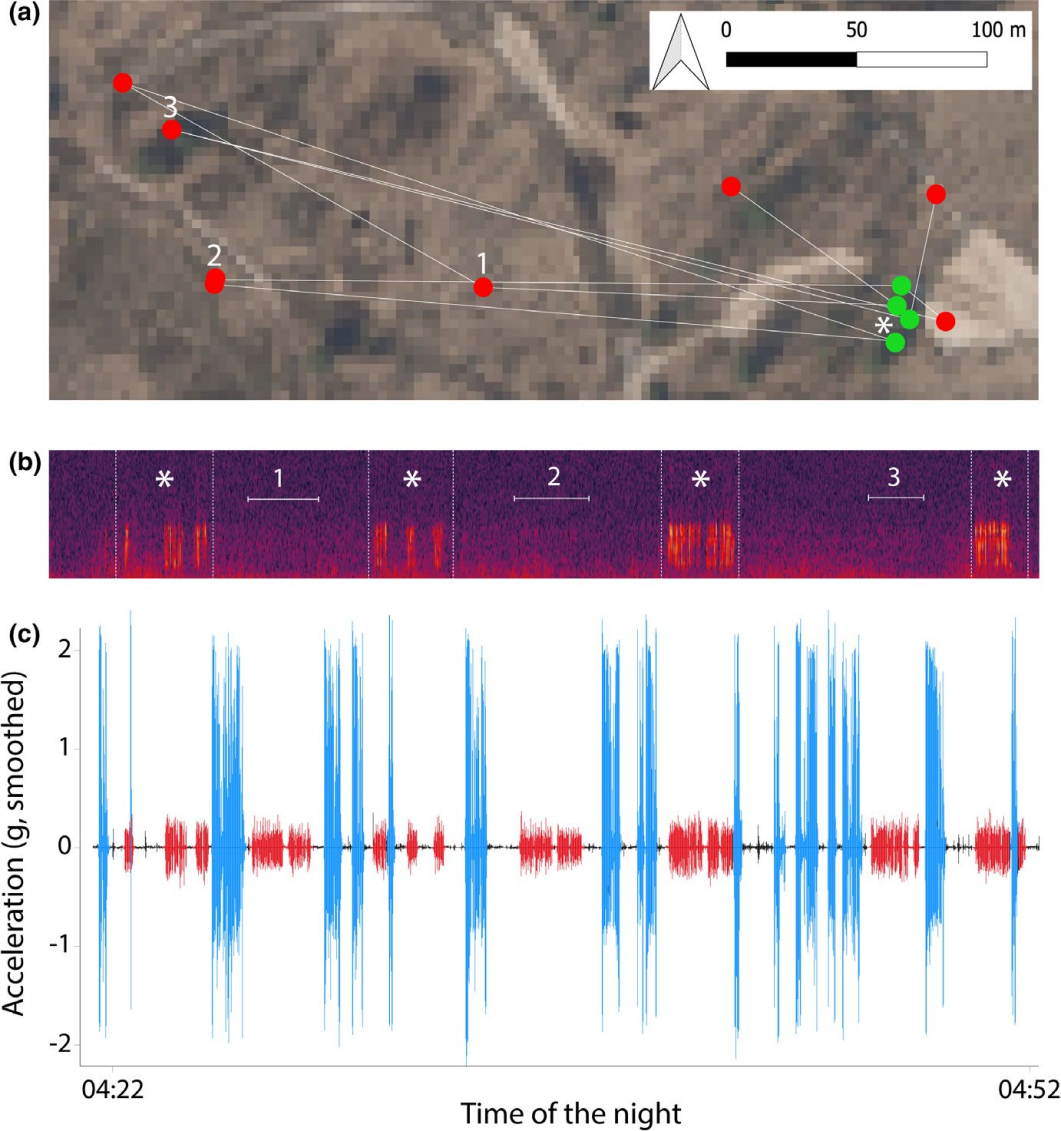
Space use, audio recordings, and singing activity of one male near one audio recorder in the same 30‐min timeframe. Space use (a) of one male (GPS locations, 3‐min interval) near one audio recorder (*). Four recorded song bouts (b: indicated with *) overlap with the male's presence near the audio recorder (a: green dots). For other GPS observations, no song bouts were recorded (a: red dots). Acceleration data (c) demonstrate singing activity during the male's presence near the audio recorder (a: green dots) which overlaps with the recorded song bouts (b: indicated with *). Additionally, singing activity (c) was observed from acceleration data, but not from audio recordings when the male was further from the audio recorder (a, b: red dots, indicated with numbers 1–3)

The crepuscular/nocturnal behavior of nightjars impedes an elaborate field study that would enable the direct annotation of acceleration measurements in relation to the species’ behavior (Bom et al., 2014; Nathan et al., 2012; Shamoun‐Baranes et al., 2012). Instead, we combined GPS data and field observations, an already widely used approach for behavioral classification and validation of these classifications in free‐living birds (Nathan et al., 2012; Patterson, Gilchrist, et al., 2019). We investigated GPS tracking data of well‐known individuals (Evens, Beenaerts, Neyens, et al., 2018; Evens et al., 2020) and we used field observations, sound recordings, and thermal videos (Pulsar Helion XQ38F Thermal Imaging Scope) to identify unique events of the target behaviors which could then be linked to acceleration measurements (Table 1; for a description of other target behavior, see Appendix S2). In case of song behavior, we focused on identifying “churring” events. Males produce the distinctive “churring” song from a stationary song post (Rebbeck et al., 2001; Zwart et al., 2014). Therefore, we investigated GPS data to differentiate between stationary periods (Table 1: resting or singing) or movement (Table 1: flying or leaping; Figure 3b: blue lines) either within or outside known breeding habitat. Stationary periods could be identified as clustered GPS observations (spatial error ± 20 m; Evens, Beenaerts, Ulenaers, et al., 2018). During these stationary periods, we expected nightjars’ body to vibrate when they were “churring.” This enabled us to distinguish between resting (i.e., low‐amplitude body acceleration; Figure 3b: black lines) and singing (i.e., medium‐amplitude body acceleration; Figure 3b: red lines).

#### 2.2.2 Modeling

We used an unsupervised machine learning approach to differentiate four nocturnal behaviors (rest, sing, fly, and leap) from one‐dimensional accelerometer data. We fitted random initializations for hidden Markov models (one continuous variable; RcppHMM R‐package; Cardenas‐ovando et al., 2017) containing four to seven hidden states to a representative training dataset. The training dataset contained 50% of the acceleration data of one night from one well‐known individual whose song posts, foraging areas, and general space use were also investigated in a previous study (Evens, Beenaerts, Neyens, et al., 2018). We then ran an expectation maximization algorithm (10,000 iterations) to estimate the model predictors based on the smoothed acceleration data. We used the Viterbi algorithm to estimate the most likely sequence of states (hereafter predicted states) to have generated from the observed acceleration measurements. We evaluated each model's performance based on a visual inspection of classification of the training dataset and opted for the five‐state model (using two states for inactive behavior) for the classification of the full dataset. We converted the predicted states to the four target behaviors by further specifying the behaviors. The additional specifications are based on behavior‐specific information extracted from literature (Cresswelll & Alexander, 1992; Evens, Beenaerts, Neyens, et al., 2018; Rebbeck et al., 2001) and observations from exclusive behavioral events. In case of singing behavior, for example, we considered bouts to be biologically meaningful if they were longer than 10 s (Rebbeck et al., 2001) and omitted song bouts shorter than 10 s. Song bouts were defined as uninterrupted classifications of singing behavior. Lastly, we subsampled all data to 1‐s intervals because the target behaviors occur at intervals which are larger than 1 s.

#### 2.2.3 Validation of song activity with audio recordings

For the purpose of this study, we only consider the validation of song activity. For one male, high‐quality song recordings were made at two of its song posts in Klein Schietveld (night from July 23 to 24). We used these song recordings to validate the accuracy of song classification by the hidden Markov model. Acceleration and audio spectrograms were visually aligned (both recorded in GMT; spectrograms were opened in R 3.6.3 and in Raven Pro 1.5.0, respectively). We distinguished between classifications of song activity for periods when the focal individual was closer/further than 20 m from the recorder (20 m is the maximum distance at which start and end time of song were clearly audible on recordings, distance tool in ArcGis 10.7.1.). The start time, end time, and duration of each song bout (acceleration data + song recording) were manually determined (±1 s, only recorded song bouts of at least 10 s). We discriminate between true‐positive song detections (song detections that could be verified based on the audio recordings), false‐positive song detections (song detections that could not be verified based on the audio recordings), and false‐negative song detections (song on audio recording that was not recognized by the model).

Since the durations of the song bouts were not normally distributed (Shapiro–Wilk *W* test, *W* < 0.9), a spearman rank correlation test was used to determine whether the durations of the audio and acceleration song detections were correlated. In order to determine whether the durations of the detected song bouts differed between the two methods (acceleration data and audio recordings), a Wilcoxon signed rank test and a paired t‐test were used; after removing song bouts with a recorded duration of <20 s, the durations were normally distributed (Shapiro–Wilk *W* test, *W* > 0.9).

## 3 RESULTS

**FIGURE 4:**
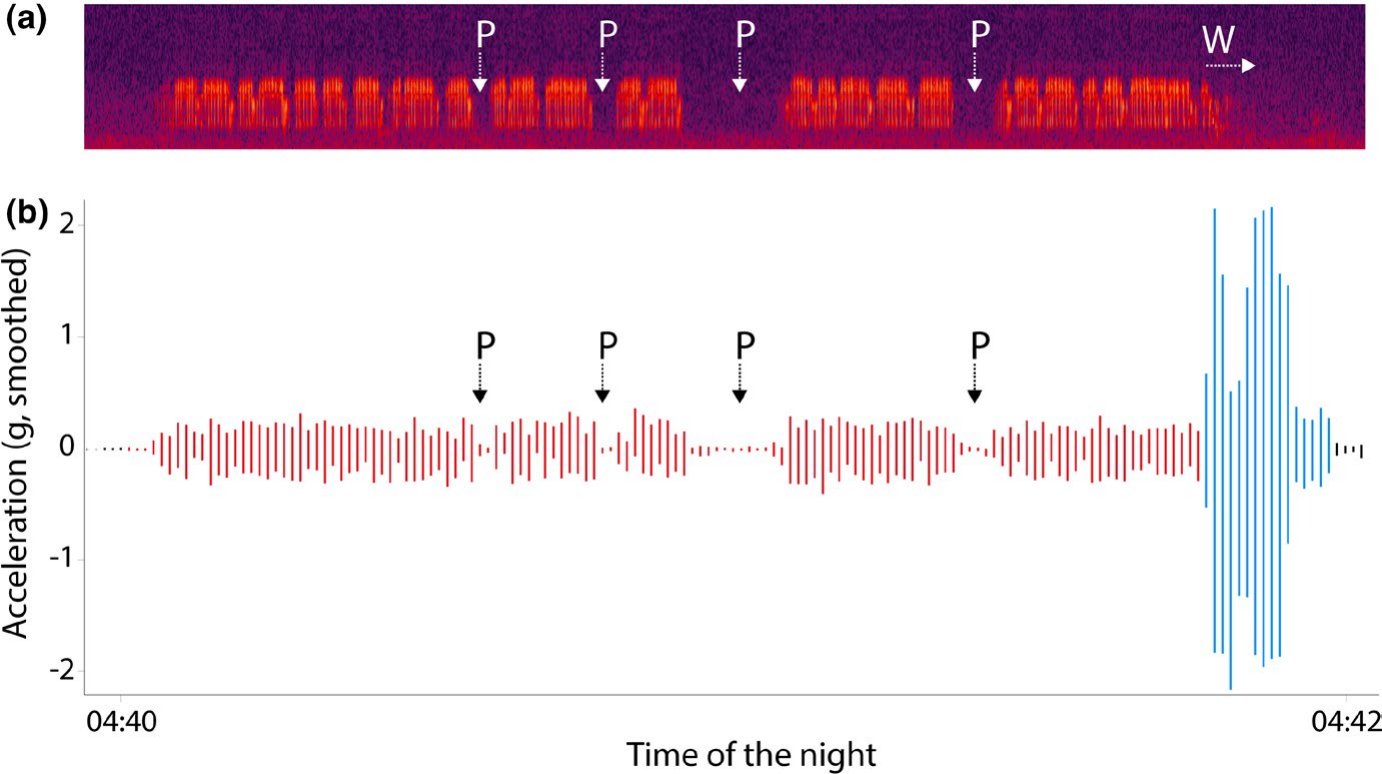
Simultaneous audio recording and acceleration data in a 2‐min timeframe. One 2‐min song recording (a) shows the alternation between song strophes, interrupted by brief pauses (P), and ending in a wing clapping phase (W). This tightly overlaps with the male's acceleration data (b). Simultaneously recorded acceleration data (b) demonstrate the same alternation between singing activity (red) and pauses (P). The terminal wing clapping phase is reflected by the acceleration data as high‐pitched flight activity (blue). See embedded Video 1

Although acceleration data were successfully collected for seven individuals, simultaneously recorded GPS and acceleration data and clear audio recordings were available only from one night for one male in Klein Schietveld, at two of its song posts. Battery changes of the audio recorders, within the 48 h data collection timeframe of accelerometers, caused an unfortunate mismatch in simultaneous recordings and measurements. A total of 68 potential song bouts of at least 10 s (median duration 25 ± 39 s; 11–204 s) were classified by the hidden Markov model. Fifty‐six potential song bouts were classified when the male was further than 20 m from one of the audio recorders and 12 potential song bouts were classified when the male was located <20 m from one of the audio recorders (Table 2; Figures 3 and 4). In the latter case, when the male was closer than 20 m from an audio recorder (Figure 3, Table 2), one of the recorded song bouts was misclassified by the model as a leaping event (false negative; Appendix S3). This means that the number of classified song bouts should have been 13, leading to a classification accuracy of 92%. Audio recorders detected 11 of the 13 classified song bouts (85%). Two song bouts, classified from the acceleration data, could not be detected on the audio recordings (Figure 3). When the male was more than 20m from the audio recorders, audio recorders only detected four song bouts (8%; 4 of 56); leaving 52 (93%; 52 of 56) potential song bouts undetected by the audio recorders (Table 2). Overall, this means that only 15 of 68 potential song bouts (22%) were detected by the audio recorder (Video 1).

**TABLE 2:** Number of song bouts detected by the model (Model), number of recorded song bouts (Audio), number of matches between the model and the recordings (Match), number of song bouts detected by the model but not recorded (Model only), and number of recorded song bouts that were not detected by the model (Audio only) at various distances from the recorders

Distance	Model	Audio	Match	Model only	Audio only
<20	12	11	10	2^a^	1
>20	56	4	4^a^	52	0
All	68	15	14	54	1

^a^ The classification model detected flight activity immediately before and/or after the singing activity, meaning that the nightjar was stationary for <3 min during singing. Therefore, it is likely that the true location of the song post was not registered by the GPS logger.

**VIDEO 1:** Animation of singing nightjar containing accelerometer data (top) and sonogram (bottom)

**VIDEO 2:** Thermal video of flying nightjar

**VIDEO 3:** Thermal video of flycatching nightjar

Further comparison of the 10 matched recorded and classified song bouts (13 [actual song bouts] – 1 [false negative song bout] – 2 [undetected song bouts] = 10 [matched song bouts]; male closer than 20 m from the audio recorders) demonstrates that the duration of these classified song bouts is significantly correlated with the recorded song bout length (correlation coefficient = 0.87, *N* = 10, *S* = 21.7, *p *= .001; Figures 3 and 4). The duration of all classified song bouts was on average 7.8 seconds longer than the duration of the simultaneously recorded song bouts (*N* = 10, *V* = 4.5, *p *= .02; Figure 4). Recorded song bouts longer than 20 s did not differ in length from classified song bouts (*N* = 5, *t* = −1.2, df = 4, *p *= .3).

## 4 DISCUSSION

Our study shows that accelerometer‐based identification of vocalizations could serve as a promising tool to study communication in free‐living, small‐sized birds. Validation of the classification model was possible when the male sang sufficiently close to an audio recorder (<20 m), indicating that song activity was detected by the classification model with an accuracy of 92%. Classified song bout length was highly correlated with that of recorded song bouts. At the same time, accelerometer data suggest that the audio recorders only captured approximately 20% of song bouts produced by the studied individual; hence, demonstrating possible limitations of such audio recorders to investigate individual‐based variation in song behavior.

The current classification accuracy of song bouts produced near an audio recorder is 92%, meaning that 10 of 11 song bouts were correctly classified from acceleration data (Figure 3), with an accurate estimate of song bout length (Figure 4). One false‐negative classification was a clear misclassification by the model, and comprised a song bout midst of a period with intensive flying (Appendix S3). Importantly, GPS data initially suggested two putative false‐positive classifications of song bouts close to an audio recorder. Here, we observed flight activity prior and after the song bouts, indicating that the individual shortly moved out of the detection range of the audio recorder and did not remain stationary at its song post for a period longer than 3 min.

Besides the identification of different song bouts, the estimated duration of song bouts was highly accurate. The model overestimates song bout length by 7.8 s in case song bouts are shorter than 20 s. The estimated duration of longer song bouts does not differ between audio recordings and model estimates. Our data were collected in the night from July 23 to 24, when the vocal activity is no longer peaking (Schlegel, 1967). Early in the breeding season (May), song bouts can last for up to 10 min and longer (own observations; Schlegel, 1967), which means that song bouts are significantly shorter in July. To what extent overall daily song activity might be overestimated later in the breeding season, therefore, remains to be investigated.

With a total weight of 3.1 g, the tag combination is appropriate to deploy on medium‐ to small‐sized birds (Rutz & Troscianko, 2013; although the impact of tags and their weight should be investigated separately for each species (Portugal & White, 2018)). While the choice for tail‐mounted devices was initially made to facilitate the retrieval of data loggers (Evens, Beenaerts, Ulenaers, et al., 2018), this alternative logger placement may have unintentionally improved the detectability of subtle body vibrations, and allowed us to discriminate between “resting” and “vocally active.” It may be interesting to investigate, test, and validate how alternative logger placements may be useful to detect vibrational signals or body movement associated with vocal communication in birds with more complex song types (Alonso et al., 2021; Gudka et al., 2019). With research primarily focusing on internal and environmental factors influencing nightjars’ “churring” song behavior, we did not investigate all types of display behavior in our study. Wing clapping and a “bubbling” song type (Figure 4) are often displayed in‐flight at the end of a nightjar song bout. These types of aerial displays, also performed by many other species such as Larks, can currently not be identified. More behavioral details may be acquired by enhancing the acceleration sampling rate to the range of the kHz and/or by using tri‐axial accelerometers combined with species‐specific observations.

Using a more sophisticated version of the accelerometers than in the current study (three‐dimensional measurements and improved battery capacity), it will be possible to quantify daily song activity of individual nightjars continuously up to 10 days/nights and determine the timing and approximate length of each song bout. This information will fulfil the requirements to study proximate and ultimate factors shaping vocal communication in birds, using nightjars as a model organism. The “churring” song of nightjars is powerful and less complex than many other bird songs, which will facilitate the investigation of new questions regarding intra‐ and inter‐individual variation in song output in response to internal (e.g., mating status, age, and body condition) and/or environmental factors (e.g., lunar cycle, weather conditions, and artificial light) that contribute to individual‐ and population‐level variation in song output. For example, to unravel the functions of song (mate attraction and/or territory defense), it is important to know how song expression varies between pairing and breeding stages (Gienapp & Merilä, 2010; Moran et al., 2019). This seasonal variation in song activity can be strongly affected by weather factors (Bruni et al., 2014; Hasan, 2010; Keast, 1994; Naguib et al., 2019; Schäfer et al., 2017) and the lunar cycle (Alonso et al., 2021; Dickerson et al., 2020; York et al., 2014). A further application can be sought in determining nightjars’ time activity budget. For instance, time‐related trade‐offs between singing and foraging can be investigated using the same recording device. Until now, mainly behaviors related to locomotion have been classified from accelerometer data, like “resting,” “flying,” “swimming,” etc. (Kays et al., 2015; Nathan et al., 2012; Patterson, Elliott, et al., 2019). Given indications that nightjars’ nocturnal flight activity (Evens, Kowalczyk, Norevik, et al., 2020), breeding ecology (Mills, 1986), and vocal activity (Reino et al., 2015) are strongly influenced by the lunar cycle, it is likely that anthropogenic influences, and especially astronomical light pollution (indirect artificial light irradiation, perceptible over large distances, especially during covered nights), can have important implications for daily behavioral trade‐offs. To address these questions, individual‐based recordings will provide much more valuable information in the near future.

We highlight that the use of accelerometers could overcome important shortcomings in the study of vocal behavior of free‐living animals. Firstly, our results suggest that only 20% of the daily song bouts are captured by the audio recorders, which were distributed over the presumed territory of the tracked male. Indeed, stationary recorders likely record only a subset of an individual's vocal output (Johnson et al., 2009) because individuals’ song posts can be distributed over a wide area, even outside the presumed territory (Evens, Beenaerts, Ulenaers, et al., 2018), impeding a complete coverage with stationary sound recorders. Secondly, animal‐borne accelerometers allow singing activity to be unambiguously attributed to the focal animal. Even when using animal‐borne microphones, it can be challenging to discriminate the vocalizations of the focal individual from those of nearby conspecifics (Anisimov et al., 2014; Gill et al., 2015; Greif & Yovel, 2019); and stationary microphones may only record individuals when they vocalize sufficiently close to the recorder. Recent studies have used autonomous, bioacoustic recorders to monitor vocal activity of nightjars at specific sites (Zwart et al., 2014). Although song activity of individual nightjars can readily be identified, identification is only reliable for a limited group of individuals (Rebbeck et al., 2001; Zwart et al., 2014). This means that studying individual vocal activity using animal‐borne devices could therefore give better insights into the determinants of vocal activity of individual nightjars. Thirdly, the use of animal‐borne accelerometers allows that individuals can be recorded undisturbed in their natural environment for prolonged periods, whereas previously, individual‐based studies on vocal behavior either struggled with short recording durations (Couchoux et al., 2015; Cvikel, Egert Berg, et al., 2015) or had to be carried out on captive animals (Gill et al., 2015, 2016; Magno et al., 2020).

With the help of new biologging devices, knowledge gaps can be filled concerning animal behavior, especially when thoughtfully combined with acoustic and visual observations (Smith & Pinter‐Wollman, 2021). Our study shows that accelerometers can serve as a cheaper, lighter, and longer‐lived alternative to microphone tags to study vocal behavior of animals with relatively simple song types. It will open new perspectives to study the vocal/display behavior in great detail and with individual‐level resolution, even in difficult to observe species. We anticipate further validation of our methods, using more sophisticated devices, in order to improve the identification of different vocalizations and support a broader application of this method.

## CONFLICT OF INTEREST

The authors declare no conflict of interest.

## AUTHOR CONTRIBUTIONS

**Elena Eisenring:** Formal analysis (lead); Investigation (equal); Validation (equal); Writing – original draft (lead); Writing – review & editing (equal). **Marcel Eens:** Conceptualization (equal); Resources (equal); Supervision (equal); Writing – original draft (equal); Writing – review & editing (equal). **Jean‐Nicolas Pradervand:** Conceptualization (supporting); Writing – review & editing (supporting). **Alain Jacot:** Conceptualization (supporting); Writing – review & editing (supporting). **Jan Baert:** Data curation (equal); Formal analysis (equal); Methodology (equal); Writing – review & editing (supporting). **Eddy Ulenaers:** Conceptualization (supporting); Investigation (supporting); Writing – review & editing (supporting). **Michiel Lathouwers:** Resources (supporting); Writing – review & editing (supporting). **Ruben Evens:** Conceptualization (lead); Data curation (lead); Formal analysis (supporting); Funding acquisition (lead); Investigation (equal); Methodology (equal); Validation (equal); Visualization (lead); Writing – original draft (supporting); Writing – review & editing (equal).

### OPEN RESEARCH BADGES

This article has earned an Open Data Badge for making publicly available the digitally‐shareable data necessary to reproduce the reported results. The data is available at https://osf.io/sem84/.

## Supporting information

Supplementary Material

## Data Availability

Data are available from the OSF Repository at: https://osf.io/sem84/.
