# Supplementary material for: Quantifying song behavior in a free‐living, light‐weight, mobile bird using accelerometers
Source: Ecol Evol. 2022 Jan 23;12(1):e8446. doi: 10.1002/ece3.8446 (PMC8803288; doi:10.1002/ece3.8446)
Supplement: Supplementary file 1 — Supplementary Material [file ECE3-12-e8446-s001.docx]

**Appendices**

Appendix S1: Overview of tagged birds.

*Table S1: Overview of tracked individuals. Duration = attachment period (days) of tracking device on bird.*

| **ID** | **Country** | **Site** | **Date** | **Latitude** | **Longitude** | **Sex** | **Age** | **Weight** | **Duration** |
| --- | --- | --- | --- | --- | --- | --- | --- | --- | --- |
| J83 | Belgium | Klein Schietveld | 22/07/2019 | 51.38 | 4.49 | M | >2CY | 72.3 | Lost |
| J46 | Belgium | Kalmthout | 01/07/2019 | 51.39 | 4.41 | M | >1CY | 70.9 | 10 |
| J36 | Belgium | Kalmthout | 17/06/2019 | 51.39 | 4.41 | M | >2CY | 65 | 3 |
| J43 | Belgium | Kalmthout | 01/07/2019 | 51.39 | 4.41 | M | >1CY | 62.5 | 8 |
| J42 | Belgium | Kalmthout | 01/07/2019 | 51.39 | 4.41 | M | >2CY | 69 | 7 |
| J79 | Belgium | Klein Schietveld | 22/07/2019 | 51.38 | 4.49 | M | >2CY | 68.5 | 6 |
| I63 | Belgium | Kalmthout | 17/06/2019 | 51.39 | 4.41 | M | >2CY | 57.1 | 3 |
| I96 | Belgium | Kalmthout | 01/07/2019 | 51.39 | 4.41 | M | >2CY | 63.2 | 10 |

Appendix S2: Extended version of behaviour classification

We designed an ethogram of the target behaviours: inactive, sing, fly and leap (Table A2). The crepuscular/nocturnal behaviour of nightjars impedes an elaborate field study that would enable the direct annotation of acceleration measurements in relation to the species’ behaviour. Instead, we investigated GPS-tracking data of well-known individuals, and we used field observations, sound recordings (SongMeters; Wildlife Acoustics) and thermal videos (Pulsar Helion XQ38F Thermal Imaging Scope) to identify events of the target behaviours which could then be linked to acceleration measurements (Appendix Table A2; for the detailed classification process).

*Table S2. Ethogram of target behaviours. Exclusive events of the four behaviours were identified from GPS-observations, validated using various types of field observations and linked with accelerometer measurements. GPS-observation: type of GPS-observation used for the identification of behaviour. Verification: information/method used to validate the GPS-observations.*

| **Behaviour** | **Locomotion** | **Description** | **GPS-observation** | **Verification** |
| --- | --- | --- | --- | --- |
| Rest | No | Standing or sitting | Clustered, daytime | Visual observations |
| Sing | No | Singing | Clustered, breeding habitat | Song recordings |
| Fly | Yes | Flying | Scattered observations | Inbound commuting flights |
| Leap | Yes | Chasing prey | Clustered, foraging habitat | Thermal videos |

*GPS-tracking data*

In previous GPS-tracking studies (Evens, Beenaerts, Neyens, et al., 2018; Evens, Jacot, Artois, Ulenaers, Neyens, Rappaz, Theux, et al., 2020), investigating the spatial use of nightjars, we showed that it is possible to distinguish whether an individual remains stationary or is moving (Evens et al. 2018). We considered stationary periods as clustered observations (spatial error ± 20m) in breeding or foraging habitats. We identified local movement in breeding or foraging habitats as scattered observations (spatial error > 20m). We recognised commuting flights as linear observations that indicate directional movement (Cresswelll & Alexander, 1992; Evens, Beenaerts, Neyens, et al., 2018).

*Identification of other target behaviours: Inactivity*

Nightjars remain motionless during most of the day. Therefore, we used information from individuals at their daytime roost as a reference for acceleration measurements related to “*inactivity*”. Visual observations confirmed the inactivity of these birds. Acceleration data related to inactivity can be recognised as low-amplitude acceleration (Appendix Figure A1A).

*Identification of other target behaviours: Flying*

During twilight nightjars spend much of their time flying within their territory and commuting between breeding and foraging sites (Wynne-Edwards, 1930; Mills, 1986; Evens et al., 2017). We identified exclusive flying behaviour from inbound flights after individuals were observed foraging in distant foraging sites as long-term, high-amplitude activity (Evens et al. 2018) (Appendix Figure A1C). The amplitude of acceleration measures related to flying is higher compared to singing, but lower compared to leaping (see below, Appendix Figure A1D and Embedded Video 2).

*Identification of other target behaviours: Leaping*

Nightjars are aerial insectivores which forage during sustained flight (aerial hawking) or when perched (flycatching) (Cresswelll & Alexander, 1992). In both foraging tactics, nightjars predominantly detect flying prey against the sky to catch them in a short, steep upward leap (Cramp, Simmons, & Perrins, 1985). To recognize this leaping behaviour from accelerometer data, we initially focussed on flycatching individuals. Flycatching can be recognized from GPS-data as clustered observations within typical foraging habitat (Evens, Jacot, Artois, Ulenaers, Neyens, Rappaz, Theux, et al., 2020) because individuals are perched and only perform short flight bouts (2-7 seconds; Cresswelll & Alexander, 1992) to catch prey from air. To observe this behaviour, we visited potential foraging sites of well-known, tagged individuals (Evens, Beenaerts, Neyens, et al., 2018; Evens, Jacot, Artois, Ulenaers, Neyens, Rappaz, & Theux, 2020) and recorded flycatching using a thermal camera (*Appendix Figure A1*D, Embedded Video 3). This allowed us to identify leaping behaviour as short-term, very-high-amplitude activity (*Appendix Figure A1*D). Leaping has a distinctively higher-amplitude acceleration compared with resting (*Appendix Figure A1*A), singing (*Appendix Figure A1*B) and flying (*Appendix Figure A1*C), which allowed a straightforward identification of this behaviour.

*
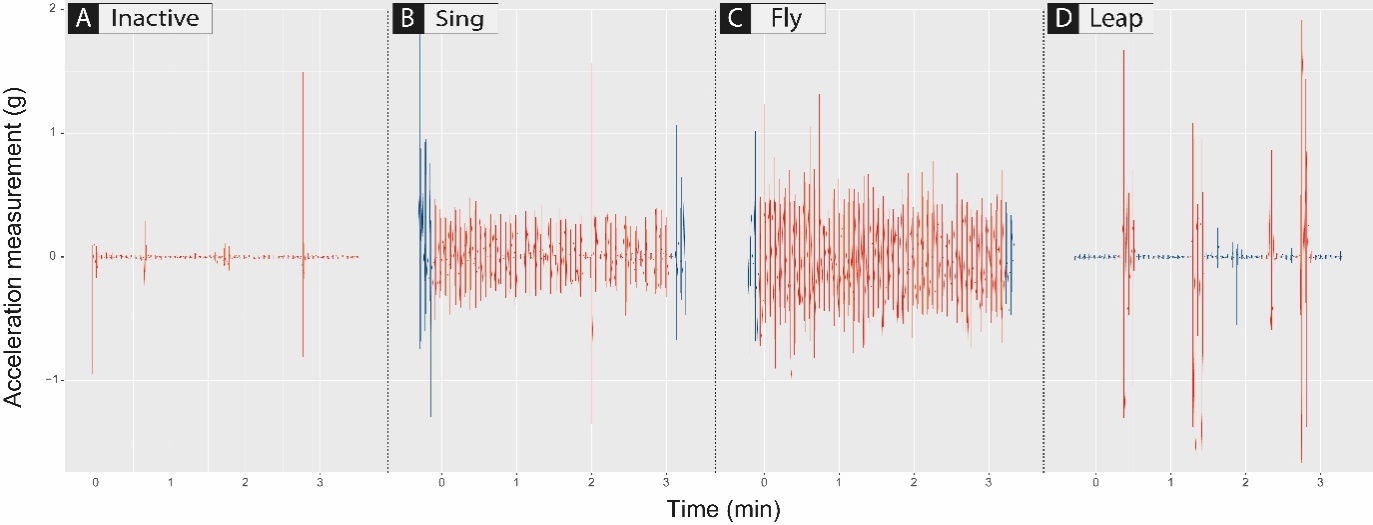
*

*Figure S1: Examples of different behaviours assigned to acceleration measurements. In all panels, acceleration in the Z-axis (heave) is shown with a red (specific behaviour of the panel) or blue line (other behaviours). Each behaviour is characterized by either low- (A. inactive), medium- (B. sing), high- (C. fly) or very high-amplitude acceleration (D. leap). Leaping is caused by short bursts of very high-amplitude acceleration, here shown as an individual performing four attempts while perching.*


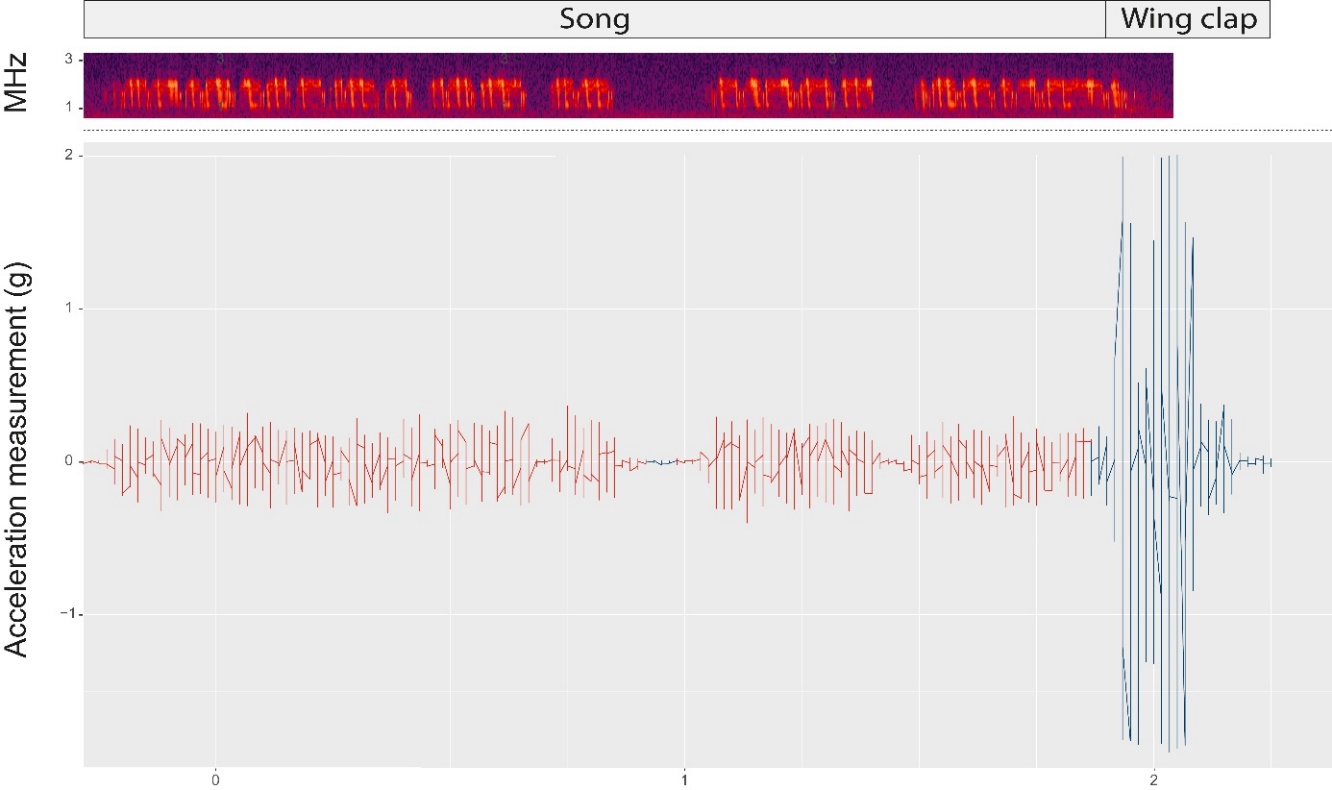


*Figure S2: Example of a song bout recorded by a SongMeter and an accelerometer. Top. A two-minute sonogram (expressed in MHz) comprising several song strophes as recorded by a SongMeter. Bottom. The same song recorded by an accelerometer shows the characteristic medium-amplitude acceleration of the nightjar’s body (red line). The song is ended with wing clapping, hence the very-high amplitude acceleration (blue line).*

Appendix S3: False negative classification


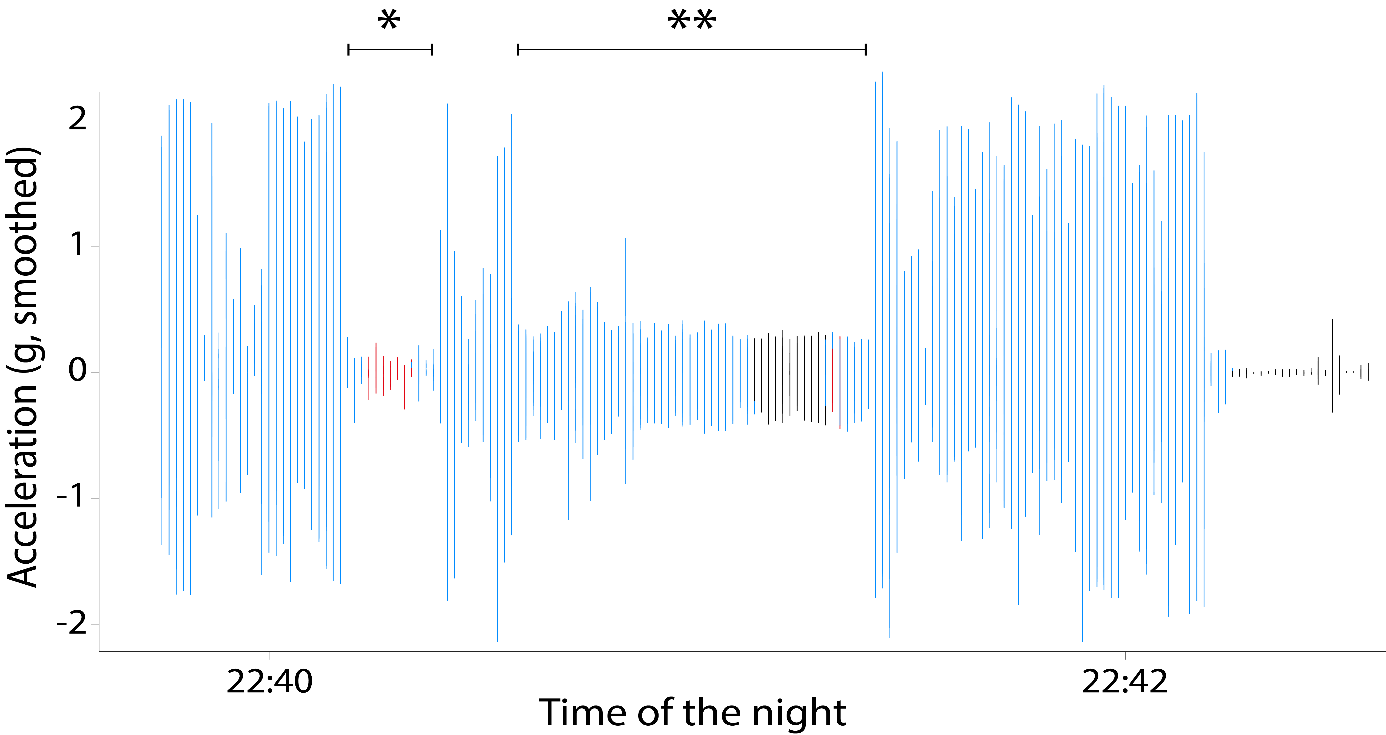


*Figure S3: Example of a misclassification of accelerometer data. Blue acceleration in the Z-axis (heave) represents flying or leaping behaviour (high and very-high amplitude acceleration). Black acceleration represents resting (low amplitude acceleration) and red acceleration shows singing (medium amplitude acceleration). The section highlighted with “ * ” is a short song bout (shorter than 10 seconds), which was not considered for the analysis. The section highlighted with “ ** “ shows the misclassification, which was -likely- not picked up by the model due to the high amount of flying and leaping in that part of the evening.*
